# Khat and alcohol use and risky sex behaviour among in-school and out-of-school youth in Ethiopia

**DOI:** 10.1186/1471-2458-5-109

**Published:** 2005-10-14

**Authors:** Derege Kebede, Atalay Alem, Getnet Mitike, Fikre Enquselassie, Frehiwot Berhane, Yigeremu Abebe, Reta Ayele, Wuleta Lemma, Tamrat Assefa, Tewodros Gebremichael

**Affiliations:** 1 Department of Community Health, Addis Ababa University, Addis Ababa, Ethiopia; 2 Department of Psychiatry, Addis Ababa University, P.O.Box 9086 Addis Ababa, Ethiopia; 3 Ethiopian Public Health Association, Addis Ababa, Ethiopia; 4 Ministry of Defence, Addis Ababa, Ethiopia; 5 Family Health International, Addis Ababa, Ethiopia; 6 Private consultant, Addis Ababa, Ethiopia

## Abstract

**Background:**

Khat (an evergreen plant with amphetamine-like properties) and alcohol are widely consumed among the youth of Ethiopia. However, their relationship to risky sexual behaviour is not well described. This study was conducted to describe the magnitude of risky sexual behaviour (unprotected sex and early initiation of sexual activity) and its association with Khat and alcohol consumption in Ethiopian youths.

**Methods:**

A probabilistic national sample of 20,434 in-school and out-of-school youths aged between 15 and 24 years of age was selected and interviewed regarding their sexual behavior and substance use.

**Results:**

Over 20% of out-of-school youth had unprotected sex during the 12-month period prior to interview compared to 1.4% of in-school youth. Daily Khat intake was also associated with unprotected sex: adjusted OR (95% CI) = 2.26 (1.92, 2.67). There was a significant and linear association between alcohol intake and unprotected sex, with those using alcohol daily having a three fold increased odds compared to those not using it: adj. OR (95% CI) = 3.05 (2.38, 3.91). Use of substances other than Khat was not associated with unprotected sex, but was associated with initiation of sexual activity: adj. OR (95% CI) = 2.54 (1.84, 3.51).

**Conclusion:**

A substantial proportion of out-of-school youth engage in risky sex. The use of Khat and alcohol and other substances is significantly and independently associated with risky sexual behaviour among Ethiopian youths.

## Background

Over 6% of Ethiopia's adult population is believed to be HIV positive. It is estimated that a large proportion of new infections occur in people aged 25 years or younger. As is the case elsewhere in Africa, transmission is almost exclusively through heterosexual contact [1], although parenteral transmission through injections account for some infections. Postponement of sex, adoption of safe sex practices, including protected sex, and prevention of parenteral transmission are the cornerstones of national and international HIV/AIDS control strategies[2]. Surveys have indicated a generally high level of awareness of HIV/AIDS[3], and increased condom use in the Ethiopian population[4]. Substance abuse is generally believed to be one of the associated factors for sexual risk behaviour in HIV transmission. Hard drugs like heroin and cocaine are very rarely available in Ethiopia. However, khat, a locally produced psycho-stimulant is commonly and widely used in the country.

Khat *(Catha edulis) *is an evergreen plant that grows mainly in Ethiopia, Yemen and other African countries along the coast of the Indian Ocean. It has been used for centuries as a mild stimulant. The fresh leaves are chewed or consumed as tea. For most youths chewing Khat is a method of increasing energy and elevating mood in order to improve work performance[5]. The psycho-stimulant effect of Khat is due to the alkaloid ingredient cathinone, which has a similar chemical structure to amphetamine[6]. Several case reports and population studies have shown that there is a clear association between heavy consumption of khat and psychosis [7-13]. Khat is widely consumed among the youth of Ethiopia as shown by several prevalence studies[14]. Although the deleterious physical effects of Khat has been shown by some studies[15], its role in altering sexual behaviour has not been studied or reported. While some attribute their sexual impotence to Khat use, others report increased libido[16]. A study assessing the magnitude of Chlamydia trachomatis and Neisseria gonorrhoeae infections together with self-reports of sexual risk behavior among youths (15–24 years old) in Addis Ababa, Ethiopia, reported that increased sexual activity was significantly associated with being male, aged 20 years or over, out-of-school status, and reported alcohol/khat consumption[17].

The importance of alcohol and substance misuse as causes of significant mortality and morbidity (particularly from injuries) and social harm (such as social disruptions from crime, underemployment and marital disharmony) have been well recognized[18]. Although the effect of alcohol and other psychoactive substances in interfering with condom use has also been studied to some extent in developed societies[19], this vital area of research has not been explored in Ethiopia. Physical injury and high-risk sexual behaviour under the influence of alcohol are common in teenagers. Alcohol-related physical injury appears closely related to patterns of alcohol consumption whereas alcohol-related sexual risk-taking is most closely associated with symptoms of depression and anxiety [20].

Young people are particularly vulnerable and are the key to the future course of the HIV/AIDS epidemic. As a group, they are an essential focus for prevention and control programs. Since most new infections are in young people, modest changes in behaviour will have a significant impact on the epidemic. Thus, they have been given higher priority in the prevention and control of HIV/AIDS in Ethiopia[21]. As both Khat and alcohol are widely consumed in these groups, description of the relationship between these substances and risky sexual behaviour would usefully guide national policy and decision making on HIV/AIDS.

The present study was undertaken to describe the magnitude of risky sexual behaviour and its association with Khat consumption and alcohol use among a national sample of in-school and out-of-school youth in Ethiopia.

## Methods

Data collection for the study was conducted between December 2001 and May 2002.

### Sampling procedures

The following criteria were used in selection of the study subjects: (1) In-school youth: aged 15–19 years, unmarried, daytime high school students attending grades 9–12 or vocational training schools. (2) Out-of-school youth: aged 15–24 years, not attending day or night school, unmarried, unemployed or employed informally. The sampling frames for selection of study subjects were prepared in consultation with the Ministry of Education, regional education bureaus and respective schools (to obtain details of classes and number of students in each grade). Probability proportional to size sampling (PPS) was used to select classes in the first stage and then systematic sampling was applied to select students in the second stage. A list of classes from each selected school with their corresponding measures of size was prepared. They were listed using the numbering system of the school so that they can be identified easily. Starting at the top of the list, we calculated the cumulative measure of size (per sex) and entered these figures in a column next to the measure of size for each class. Using an average cluster size, and equal sample size for males and females, we calculated the sampling interval (SI) by dividing the total cumulative measure of size by the number of classes to be selected.

We then selected a random starting number (RS) between 1 and SI and compared this number with the cumulated measure of size column. The unit within whose cumulated measure of size for the RS falls is the first sampling unit and subsequent classes were selected by adding the SI to the RS.

Once inside the classroom, starting from the front right hand seat of the class, we picked a random number and systematically selected the required number of students. Then the selected students were requested to meet the trained interviewers at the school compound where privacy is maintained; when this was not convenient for the respondents the interviewer made an appointment to meet the student, at any suitable time and place.

Out-of-school youth (OSY) were selected from urban centres in each of the ten regions of Ethiopia and Addis Ababa and the Dire Dawa cities. The sampling frames for selection of OSY were prepared using the 1994 census report[22]. Segmentation method was used for larger regions and cities that had 20 or more Kebeles (sub-districts), and it is the preferred sampling frame for household surveys. This approach had several advantages; notably, the household had already been mapped and numbered within enumeration areas and moreover, the areas had population sizes associated with them that could be used as measures of size during sample selection, making control of the fieldwork easier. Using records from each Kebele office that contain number and size of households, we created segments for each Kebele. For instance if a Kebele has 1000 households and a sample of 100 households are needed for the survey, dividing 1000 by 100 = 10 segments were created for this particular Kebele. All segments in each Kebele had the same number of households. In randomly selected segments targeted number of households was visited, until the required number of youth was identified for interview in each segment. Each interviewer had a short checklist to determine eligibility of respondents. A face-to-face household interview was conducted to obtain the needed information. If the identified respondent was not available on the day of visit to a household appointments were made to return for the interview. A further detailed description of the sampling method is given elsewhere[23]

### Data collection and processing

Data collection was done using a standardized pre-coded and pre-tested questionnaire. Male and female interviewers were selected from each of the participating regions. Interviewers had completed high school and had some previous experience of collecting survey data. They were given a one week intensive training about the interview processes and on how to administer the questionnaire. Pilot-testing was carried out in Addis Ababa where they took the training before the interviewers were sent back to their respective sites.

Full-time editors scrutinised all completed interview forms for completeness, accuracy and consistency in the field. SPSS was used for both univariate and bivariate analysis.

History of substance use (alcohol, Khat, and others) in the last four weeks preceding the interview and history of unprotected sexual practice and initiation of sex were obtained using the interview instrument. Unprotected sex was defined as sex without the use of condom during the 12-month period preceding the interview. Irregular use of condom was also categorized as unprotected sex. Initiation of sexual activity was analysed for those younger than 20 years and was defined as a report of any sex encounter in the past. Logistic regression was employed to adjust for confounding. Thus, either unprotected sex or sex initiation was included in the logistic model as a dependent variable. As independent variables, the following were included in the model: sex, age, school status (in or out of school), educational attainment, khat, alcohol and other substance use.

Ethical clearance for the study was obtained from a national ethics committee and from Human Subjects Committee of Family Health International – USA. Participation of respondents was strictly on voluntary basis. Informed consent was solicited orally. Measures were taken to ensure the respect, dignity and freedom of each individual participating in the study. Measures were also taken to assure confidentiality.

## Results

A total of 20,434 youth aged between 15 and 24 years were included in the study. Of these, 14,224 were out-of-school youth. The response rate was 91.2% and 94.1% for out of school and in-school youth, respectively. Approximately equal number of males and females were included in the study. (Table 1). Over 80% had attained higher education, and 62% were orthodox Christians. Among in-school youth, over 90% did not use Khat or alcohol, while among the out-of-school youth close to 73% did not use Khat or alcohol. Over 23% of out-of-school youth used Khat every day or once weekly while only 7.5% of in-school youth did so. Only 0.7% of the in-school youth reported use of substances other than Khat, compared to 5.1% for out-of-school youth.

**Table 1:** Socio-demographic characteristics of the study population of in-school and out-of-school youth, Ethiopia 2003

Characteristics	Number (percent)*		
	In-school	Out-of-school	Total
*Sex*			
Male	3,089 (49.7)	7,109 (50.0)	10,198 (49.9)
Female	3,121 (50.3)	7,115 (50.0)	10.236 (50.1)
			
*Age*			
15–19 years	6,206 (100.0)	7,267 (51.1)	13,473 (66.6)
20–24 years	-	6,955 (48.9)	6,955 (34.0)
			
*Educational level*			
Not literate	-	-	-
Elementary	-	3,149 (29.1)	3,149 (18.5)
Secondary or above	6,210 (100.0)	7,687 (70.9)	13,897 (81.5)
			
*Religion*			
Orthodox Christian	4,025 (65.1)	8,648 (61.1)	12,673 (62.3)
Catholic	61 (1.0)	375 (2.6)	436 (2.1)
Protestant	351 (5.7)	1,310 (9.2)	1,661 (8.2)
Muslim	1,691 (27.3)	3,709 (26.2)	5,400 (26.5)
Other	57 (0.9)	122 (0.9)	179 (0.9)
			
*Alcohol intake*			
None or occasional	5,650 (91.1)	9,992 (73.0)	15,642 (78.7)
On a weekly basis	526 (8.5)	3,307 (24.2)	3,833 (19.3)
On a daily basis	26 (0.4)	384 (2.8)	410 (2.1)
			
*Khat intake*			
None	5,611 (91.5)	10,500 (73.9)	16,111 (79.2)
Less than one per week	67 (1.1)	364 (2.6)	431 (2.1)
Once a week	353 (5.8)	1,885 (13.3)	2,238 (11.0)
Every day	103 (1.7)	1,459 (10.3)	1,562 (7.7)
			
Substance use other than Khat**			
No	6,164 (99.3)	13,496 (94.9)	19,660 (96.2)
Yes	46 (0.7)	728 (5.1)	774 (3.8)

**Total**	**6,210 (100.0)**	**14,224 (100.0)**	**20,434 (100.0)**

* Missing values not shown.

** Substance use other than Khat include: Shisha, Benzene, Hashish, Mandrax, Cocaine, or Crack.

A total of 16,606 (82%) youth reported on their sexual behaviour and were included in the analysis (Table 2). Over 20% of out-of-school youth had unprotected sex during the 12-month period prior to the interview compared to 1.4% in-school youth. The odds of unprotected sex were slightly higher among males compared to females. Larger effect sizes were, however associated with younger age: In those aged 15–19 years compared to those age 20–24 years, adjusted OR (95% CI) = 2.54 (2.29, 2.81). Being out-of-school was strongly associated with self-report of unprotected sex: adjusted adj. OR (95% CI) = 8.48 (6.66, 10.8). Daily Khat intake was also associated with unprotected sex: adj. OR (95% CI) = 2.26 (1.92, 2.67). There was a significant and linear association between alcohol intake and unprotected sex (P-value for trend <0.01) with those using alcohol daily having a three fold increased odds compared to those not using it: adj. OR (95% CI) = 3.05 (2.38, 3.91). Use of substances other than Khat was not associated with unprotected sex.

**Table 2:** Socio-demographic and behavioral correlates of unprotected sex (during the 12 months prior to the interview) among the youth (aged 15–24 yrs), Ethiopia 2003

Characteristics	Total population	Reported unprotected sex (%)	Crude odds ratio (95% confidence interval)	Adjusted odds ratio* (95% confidence interval)	P-value
*Sex*					
Female	8,234	1,038 (12.6)	1.00 (Reference)	1.00 (Reference)	
Male	8,372	1,338 (16.0)	**1.32 (1.21, 1.44)**	**1.13 (1.02, 1.26)**	**0.02**
					
*Age*					
15–19 yrs	11,738	857 (7.3)	1.00 (Reference)	1.00 (Reference)	
20–24 yrs	4,862	1,519 (31.2)	**5.77 (5.26, 6.33)**	**2.54 (2.29, 2.81)**	**<0.001**
					
*School status*					
In-school	5,502	75 (1.4)	1.00 (Reference)	1.00 (Reference)	
Out-of-school	11,104	2,301 (20.7)	**18.9 (15.0, 23.9)**	**8.48 (6.66, 10.8)**	**<0.001**
					
*Alcohol intake*					
None	13,004	1,322 (10.2)	1.00 (Reference)	1.00 (Reference)	
On a weekly basis	2,838	852 (30.0)	**3.79 (3,44, 4.18)**	**2.02 (1.81, 2.25)**	**<0.001**
On a daily basis	318	148 (46.5)	**7.69 (6.13, 9.66)**	**3.05 (2.38, 3.91)**	**<0.001**
					
*Khat intake*					
None	13,438	1,362 (10.1)	1.00 (Reference)	1.00 (Reference)	
Occasional	342	114 (33.3)	**4.43 (3.52, 5.59)**	**2.42 (1.86, 3.13)**	**<0.001**
On a weekly basis	1,633	481 (29.5)	**3.70 (3.28, 4.18)**	**2.06 (1.79, 2.36)**	**<0.001**
On a daily basis	717	414 (36.6)	**5.12 (4.48, 5.85)**	**2.26 (1.92, 2.67)**	**<0.001**
					
Substance use other than Khat					
No	16,034	2,147 (13.4)	1.00 (Reference)	1.00 (Reference)	
Yes	572	229 (40.0)	**4.32 (3.63, 5.14)**	1.19 (0.97, 1.47)	

**Total**	**16,606**	**2,376 (14.3)**			

* Terms included in the logistic model were: sex, age, school status, alcohol intake (3 levels), Khat intake (4 levels), and substance use other than Khat.

** Substance use other than Khat include: Shisha, Benzene, Hashish, Mandrax, Cocaine, or Crack. (Shisha is a mixture that may include tobacco, honey, hashish and spices and is smoked from an oriental tobacco pipe)

Initiation of sexual activity was studied on a subset of the study population who were younger than 20 years of age. Of the total of 13,473 youths in this younger age group, 11,737 (84%) reported initiation of sexual activity and were included in the analysis. Being male or female was not associated with initiation of sexual activity when school status and substance use were adjusted for in the logistic regression model (Table 3). The odds of initiation of sexual activity were 3.6 fold higher among out-of-school youth compared to in-school youth: adj. OR (95% CI) = 3.57 (3.09, 4.12). Khat use was strongly associated with initiation of sexual activity with four-fold increased odds in both daily and weekly users. The odds ratios for daily use were: adj. OR (95% CI) = 4.13 (3.26, 5.23); and for weekly Khat use: 4.18 (3.50, 4.99). Alcohol use was strongly and linearly associated with initiation of sexual activity, those using alcohol having a four-fold increase, and daily users having six-fold increased odds of sex initiation (P for trend < 0.001). Use of substances other than Khat was also strongly associated with sex initiation: adj. OR (95% CI) = 2.54 (1.84, 3.51).

**Table 3:** Socio-demographic and behavioral correlates of initiating sex among in-school and out-of-school youth aged 15–19 yrs, Ethiopia 2003

Characteristics	Total population	Reported sexual initiation (%)	Crude odds ratio (95% confidence interval)	Adjusted odds ratio* (95% confidence interval)	P-value
*Sex*					
Female	5,932	620 (10.5)	1.00 (Reference)	1.00 (Reference)	
Male	5,806	960 (16.5)	**1.70 (1.52, 1.89)**	1.12 (0.98, 1.27)	
					
*School status*					
In-school	5,498	286 (5.2)	1.00 (Reference)	1.00 (Reference)	
Out-of-school	6,240	1,294 (20.7)	**4.76 (4.17,5.44)**	**3.57 (3.09, 4.12)**	**<0.001**
					
*Alcohol intake*					
None or occasional	9,944	939 (9.4)	1.00 (Reference)	1.00 (Reference)	
On a weekly basis	1,407	542 (38.5)	**6.01 (5.29, 6.82)**	**3.81 (3.31, 4.39)**	**<0.001**
On a daily basis	115	71 (61.7)	**15.5 (10.6, 22.6)**	**5.75 (3.70, 8.93)**	**<0.001**
					
*Khat intake*					
None	10,209	931 (9.1)	1.00 (Reference)	1.00 (Reference)	
Occasional	185	92 (49.7)	**9.86 (7.33, 13.3)**	**4.99 (3.56, 7.00)**	**<0.001**
On a weekly basis	840	335 (39.9)	**6.61 (5.67, 7.71)**	**4.18 (3.50, 4.99)**	**<0.001**
On a daily basis	445	210 (47.2)	**8.91 (7.31, 10.9)**	**4.13 (3.26, 5.23)**	**<0.001**
					
Substance use other than Khat**					
No	11,498	1,423 (12.4)	1.00 (Reference)	1.00 (Reference)	
Yes	240	157 (65.4)	**13.4 (10.2, 17.6)**	**2.54 (1.84, 3.51)**	**<0.001**

**Total**	**11,738**	**1,580 (13.5)**			

* Terms included in the logistic model were: sex, age, school status, alcohol intake (3 levels), Khat intake (4 levels), and substance use other than Khat.

** Substance use other than Khat include: Shisha, Benzene, Hashish, Mandrax, Cocaine, or Crack.

## Discussion

Our findings show that Khat and alcohol use, sex, age, and school-status were independently associated with both self-report of unprotected sexual intercourse and initiation of sexual activity. The results are unlikely to be biased. Selection of the study population used a probabilistic sampling method and was not based on any of the factors under study. Thus, selection bias is unlikely. Although information bias due to a differential and systematic under-reporting of sexual behaviour among the various variables is a possibility, it is more likely that this under-reporting is randomly distributed than otherwise. If this is the case, the resulting random misclassification will tend to bias the odds ratio towards the null value [24]. The implication for our study is that the odds ratios reported are actually underestimates of the true effect sizes in the population. We have adjusted for potential confounding by using a multivariate logistic model.

Use of alcohol, Khat and other substances were measured over the four weeks prior to the interview, while that of risky sexual behaviour over the 12 months prior to the interview. It is assumed that the four-week window assessment period is representative of long-term individual pattern of use of these substances. The correctness of this assumption can not be verified from the present study.

In this study, the association between male sex and unprotected sex, although significant, was small when other factors such as age, school status, and substance use behavior were adjusted for in a logistic model. Although hormonal factors might be expected to increase impulsiveness and risk taking behavior in males, the association with male sex may also be mediated through other intermediate behaviors such as alcohol use. In male attendees at sexually transmitted disease clinics in southern Vietnam, being aged under 20, not married, not having a current girlfriend, using alcohol before sex and substance use were all factors independently associated with visiting a female sex worker (FSW) [25]. Similarly in Colombia, a longitudinal survey revealed that adolescents with increased drug use were more likely to engage in unprotected sex as well as multiple partnerships[26].

It is more difficult to speculate on the reasons for the fairly strong two-fold association between increasing age and unprotected sex. This finding goes against previous studies and our expectation. Although we have adjusted for the possible effects of alcohol and khat use, we can not exclude the effect of other potential confounding factors.

We have also shown that being out of school was strongly associated with unprotected sex. A similar finding was reported in a previously conducted study among Addis Ababa youths, where being out of school showed a strong association with sexual risk behaviour[17]. In New South Wales, 16-year old out-of-school adolescents had consistently higher rate of reported substance use compared to their age matches in school [27]. Although underlying behavioural problems or mental disorders could be linked to the reasons for youths being out of school, and although we have previously shown that mental and behavioural disorders are prevalent in adolescents in Ethiopia[28,29], we can not confirm the association from the present study. In Bonomo et al.'s [20] study of Australian 16–17-year-olds, alcohol-related sexual risk-taking, psychiatric morbidity and high frequency of alcohol consumption had strong independent associations. In a ten-year prospective study, others reported that adolescents who have experienced drinking alcohol even once or twice during the past 12 months were more likely to exhibit more substance use, face academic problems and become involved in delinquent behaviors during the latter stages of school (middle and high school) compared to non-drinkers [30]. Early drinking was also found to be associated with early sexual initiation [31].[32]

Khat is primarily used for its stimulant effect. Users report that Khat intake results in increased energy levels and alertness, improves self-esteem, creates a sensation of elation, enhances imaginative ability and the capacity to associate ideas, and improves the ability to communicate. It has not yet been associated with alteration of rational decision-making and has not been shown to increase risk-taking behaviour[5]. Although some users also take alcohol after Khat to counteract the stimulant properties and facilitate sleep, this should not confound the observed association as alcohol use was adjusted for in the logistic model.

Our finding of a linear and strong association between unprotected sex and alcohol is to be expected because of the nature of alcohol in decreasing inhibitions, altering rational decision making, and increasing risk-taking behaviour. A qualitative study among military conscripts in Northern Thailand drew the following conclusions regarding alcohol consumption and inconsistent condom use: Alcohol is (1) consciously used by men to reduce inhibitions that constrain their interpersonal interaction with women and with each other; (2) reduces inhibitions of individuals to sexual risk taking; (3) provides a socially acceptable excuse for non-use of condoms; (4) is associated by conscripts with brothel attendance; and (5) is seen to enhance male sexual pleasure, in contrast to condoms, which are said to reduce pleasure[33]. In a meta-analysis of the association between alcohol intake and condom use, drinking at first intercourse was associated with decreased condom use, but alcohol was not related to condom use in recent sexual encounters and in recent encounters with new partners. On the other hand drinking was related to non condom use among adolescents [19].

Initiation of sexual activity was not associated with being male when other factors were adjusted in the logistic model. Others have reported that males become sexually active at a younger age than females [34]. The reason why we did not detect any difference between males and females in initiation of sex is probably due to the influence of factors other than individual behavior. For example, although in most cultures females are expected not to initiate sex before they are married, they may be forced to have sex as has been shown by recent studies of sexual abuse in Ethiopia [35, 36]. Sex initiation was also associated with being out of school. As described above for unprotected sex, the reason for the association could be underlying behavioural problems that predispose youth to leave school and also engage in risky sexual behaviour. Thus, being out of school is likely to be just a marker of such underlying behavioural and mental problems.

Khat use was also strongly associated with sex initiation. However, Khat use was assessed over the four weeks prior to the interview, while initiation of sex was assessed for a longer period spanning from puberty to 19 years of age. It is thus possible that sex was initiated well before the initiation of Khat use or vice versa. As we do not have the pattern of use of Khat beyond the four-week period prior to the survey, it is difficult to speculate on the significance of this strong association. As is the case with Khat use, the strong and linear association of alcohol use with initiation of sex, and the association of use of substances other than Khat with sex initiation, are difficult to interpret because of the uncertain temporal relationships between the various variables.

## Conclusion

This study has shown that a substantial proportion of out-of-school youths engage in risky sexual behaviours and that the use of Khat, alcohol and other substances is significantly and independently associated with risky sexual behaviour among these young people. HIV/AIDS prevention and control programmes targeting youths, should take into account this newly demonstrated association of risky sexual behaviour with use of Khat, given its widespread use among in-school and out-of-school youths, and formulate appropriate interventions to limit its use.

## Competing interests

The author(s) declare that they have no competing interests.

## Authors' contributions

TA, TG and RY were supervisors of data collection (field data quality control) and then significantly involved in the analysis and write up.

YA, involved in proposal writing, design, implementation of project and national dissemination of the findings.

WL, contributed in the designing of the methodology and all stages of the project.

FB, project manager and involved in all stages of the project implementation and write up.

GM, lead investigator and involved in mapping, designing, implementation, analysis of data, write up and dissemination.

DK, project initiator and lead investigator involved in project proposal, design of questionnaires, recruitment and training of supervisors and data collectors. He did most of the analysis and write up of the paper.

AA, investigator, involved in designing, analysis and write up.

FE, designing of the methodology and sampling procedures, coordinated mapping (listing) of study sites and target groups and managed data quality and write up of the methodology.

## Pre-publication history

The pre-publication history for this paper can be accessed here:


